# Modeling of Tumor Progression in NSCLC and Intrinsic Resistance to TKI in Loss of PTEN Expression

**DOI:** 10.1371/journal.pone.0048004

**Published:** 2012-10-24

**Authors:** Gholamreza Bidkhori, Ali Moeini, Ali Masoudi-Nejad

**Affiliations:** 1 Laboratory of Systems Biology and Bioinformatics (LBB), Institute of Biochemistry and Biophysics, University of Tehran, Tehran, Iran; 2 Department of Algorithms and Computation, College of Engineering, University of Tehran, Tehran, Iran; Howard University, United States of America

## Abstract

EGFR signaling plays a very important role in NSCLC. It activates Ras/ERK, PI3K/Akt and STAT activation pathways. These are the main pathways for cell proliferation and survival. We have developed two mathematical models to relate to the different EGFR signaling in NSCLC and normal cells in the presence or absence of EGFR and PTEN mutations. The dynamics of downstream signaling pathways vary in the disease state and activation of some factors can be indicative of drug resistance. Our simulation denotes the effect of EGFR mutations and increased expression of certain factors in NSCLC EGFR signaling on each of the three pathways where levels of pERK, pSTAT and pAkt are increased. Over activation of ERK, Akt and STAT3 which are the main cell proliferation and survival factors act as promoting factors for tumor progression in NSCLC. In case of loss of PTEN, Akt activity level is considerably increased. Our simulation results show that in the presence of erlotinib, downstream factors i.e. pAkt, pSTAT3 and pERK are inhibited. However, in case of loss of PTEN expression in the presence of erlotinib, pAkt level would not decrease which demonstrates that these cells are resistant to erlotinib.

## Introduction

The receptor tyrosine kinase (RTK) superfamily subclass I, comprises of ERBB (erythroblastic leukemia viral (v-erbb) oncogene homolog) receptors and includes four types: ERBB4, ERBB3, ERBB2 and ERBBI, the latter of which is also called EGFR (epidermal growth factor receptor) [1]. EGFR is a 170 KD transmembrane glycoprotein exhibiting enzymatic activity as a tyrosine kinase [2], [3]. The role of EGFR is to regulate some of the cellular pathways in which a ligand interacts with EGFR such as transforming growth factor-α (TGFα) and EGF ligands. EGF ligands control some of the fate-determining events in mammalian cells such as proliferation and survival which are regulated by one of the most important pathways i.e. EGFR signaling pathway [4], [5].

EGFR inhibition by means of various types of blocking agents has proved to trigger apoptosis, decrease proliferation and block angiogenesis in cancerous lung cells [6], [7]. Lung cancer is the main agent of cancer life claims in the west and is not easily diagnosed [8], [9]. No more than 15% of patients sustain life for at most five years [9]. Lung cancer is classified as two groups i.e. small-cell lung cancer (SCLC) that involves 20% of lung cancers and non-small-cell lung cancer (NSCLC) that involves 80% of lung cancers. NSCLC is believed to initiate from lung epithelial cells which leads to numerous histological sub varieties including adenocarcinoma, bronchioalveolar carcinoma, anaplastic cell carcinoma, large cell carcinoma and squamous cell carcinoma [10], [11].

Several studies have shown that the EGFR expression level enhancement is very common in the NSCLCs. EGFR concentration has been compared within several wild and cancerous lung cells [12], [13]. Over expressed levels of the EGFR have been reported in neck and head, colon, lung, breast, stomach, bladder, oesophagus, cervix, ovary and endometrium cancers which repeatedly appear to denote cancer prediction [14], [15]. EGFR over expression is abundant in NSCLC and has a correlation with the amplified gene copy number per cell. EGFR expression is not related to age, smoking, gender, pathogenic stage or tumor status. Considerable discrepancies were associated with histological differentiation in a way that highly differentiated tumor cells showed increased levels of EGFR in comparison with less-differentiated tumor cells [16]. No considerable contradiction in EGFR amounts was observed between adenocarcinomas and squamous cell carcinoma in a number of studies. However, in some other studies, the mean level of EGFR amounts was more in squamous cell carcinoma [12], [16].

It is determined that mutations in EGFR are accompanied with an elevated count of EGFR gene copies. This will result in an increased propensity of the procedure which leads to genomic loss of stability [17]. The whole kinase domain is coded with exons 18–24 and EGFR kinase domain mutations target four exons (18–21) that encode a moiety of the tyrosine kinase domain and are gathered in the vicinity of the ATP-binding site of the enzyme [18]–[23]. EGFR mutations in kinase domain are generally known as activating mutations because they seem to set off augmented kinase activity of the receptor. Nonetheless it doesn’t mean that these mutated EGFRs are completely active since the degree of their independency to the ligand might be a function of the empirical framework [24]–[26].

EGFR mutations are divided into two groups of drug resistant and drug sensitive mutations (the drugs are erlotinib and gefitinib). More than 90% of mutations are drug sensitive [27], 45% of which are in exon 19 and 40–45% occur in exon 21. Two of the most common mutations are Δ747-P753 that arises in exon 19 and L858R and take place in exon 21 [28], [29]. It has been demonstrated that ligand induced EGFR phosphorylation kinetics between wild type and mutant EGFR are different [17], [30], [31]. For example wild type receptor is phosphorylated much faster than the mutant one [17]. In addition, it is evident that activation-kinetics of downstream factors such as ERK [17], Akt [24] and STAT3/5 [24], [32] are different as well.

Our main goal was to compare the kinetics of EGFR signaling between normal and NSCLC model. Moreover, we planned to show that ERK (MAPK), STAT and Akt factor’s activation pattern are different between normal and NSCLC models. In addition we set the goal to demonstrate that loss of PTEN expression causes intrinsic resistance to TKI (tyrosine kinase inhibitors). For this reason, based on experimental data and prior corresponding models, two new expanded models were reconstructed for normal and NSCLC cells. Our results were used for evaluation and comparison of three different pathways regarding EGFR signaling, namely Ras/ERK, PI3K/Akt and STAT activation pathways.

## Materials and Methods

### Model Description

**Figure 1:**
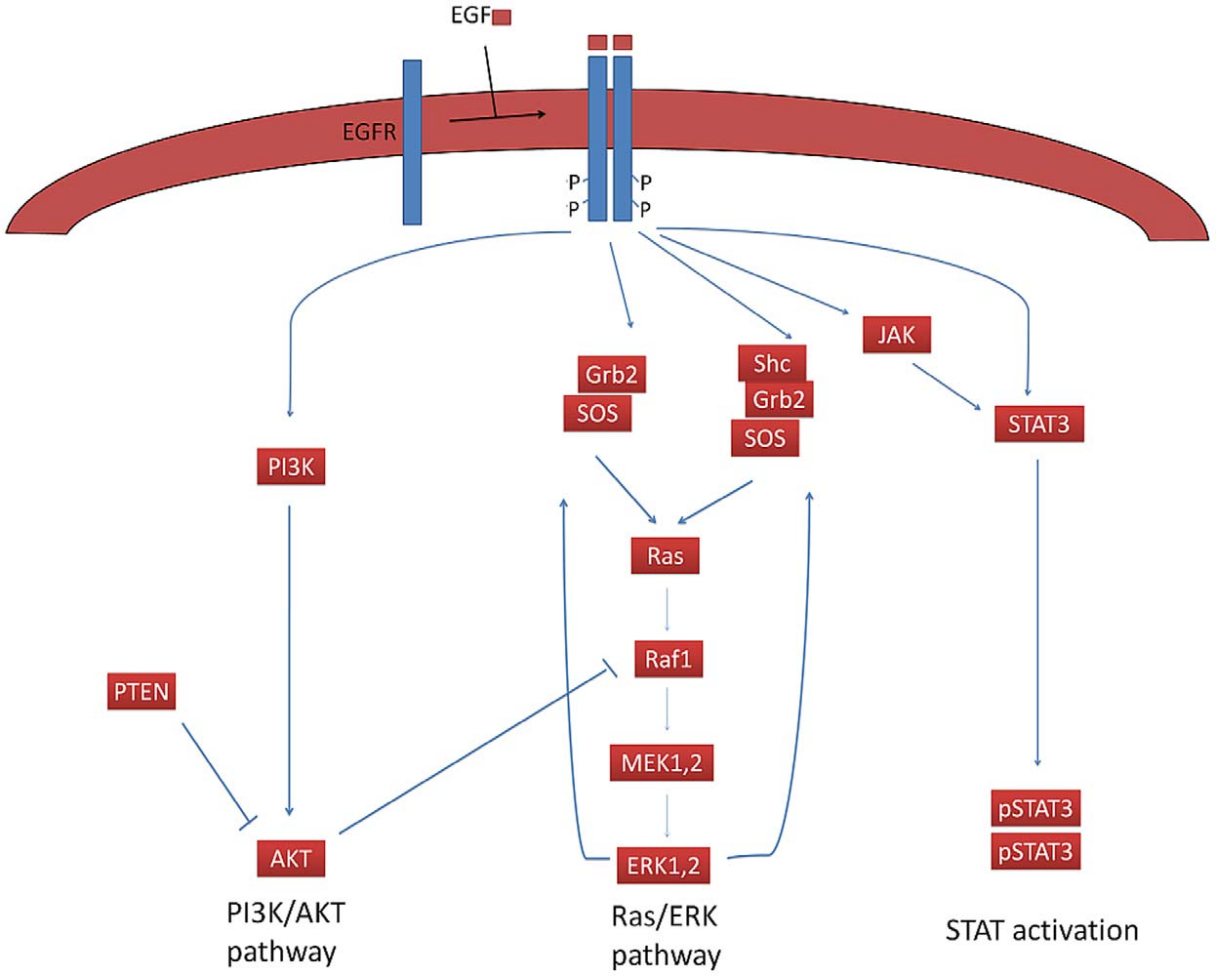
Schematic overview of the EGFR signaling. For more information refer to the context.

The desired network for basic or normal cell (normal model) was generated according to previous models and experimental observations that dealt with published EGFR signaling network dynamic behavior [33]–[41] (table S1). The network consists of three main pathways (figure 1) that play an important role in cell proliferation and survival.

These pathways are activated by EGF attachment to EGFR. After the interaction of ligand (EGF) with the extracellular domain of EGFR, the receptors undergo homo- or heterodimerization that causes auto phosphorylation of certain tyrosine residues in the cytoplasmic end, namely pY992, pY1068 and Y1173 [42], [43]. Grb2 (Growth factor receptor-bound protein 2) binds phosphorylated tyrosines (pY1148, pY1086) and Shc (Src homology 2 domain containing transforming protein) binds pY1148 and pY1173 [44]. STAT3 and STAT5 binding sites are pY1068 and pY845 [24], [45]. After C-terminal tail phosphorylation, the Shc adaptor binds its site and Grb2 attaches to Shc, and then SOS (GTP exchange protein for Ras) is employed by Grb2 [46]–[48]. Also, Grb2 directly binds its receptor, accordingly SOS binds Grb2. [49], [50]. In the next step SOS converts Ras-GDP into Ras-GTP which is the activated form of Ras [46]–[48]. Activated Ras induces Raf phosphorylation and activation [3]–[5]. In this model, Ras is treated as virtual protein kinase for Raf. Ras-GTP binds Raf and activates it. There are three kinds of Raf in cells: Raf-1(C-Raf), A-Raf and B-Raf [51], [52]. Raf is a kind of Serin/Threonine kinase that phosphorylates and activates the MEK (MAP kinase kinase). The activated MEK, phosphoralates and activates ERK1 (extracellular signal-regulated kinase 1) and ERK2 [53]–[56]. ERK or MAPK (mitogen activated protein kinase) activation triggers off some important feedback loops that exert important effects on this pathway. ERK1 and ERK2 phosphorylate a variety of the proteins that leads to cell growth and proliferation [57]–[59].

PI3K (phosphatidylinositol-3-kinase) has two 85 and 110 KD subunits which are regulatory and catalytic subunits, respectively [60], [61]. After EGFR phosphorylation, the regulatory subunit binds its phosphorylated Thyrosine site and then the catalytic and the regulatory subunits join together. Under such conditions the, PI3K is activated and converts the membrane phosphatidyl inositol 4,5bisphosphate (PIP2) into phosphatidyl inositol 3,4,5-3phosphate(PIP3). PIP3 causes Akt activation in a way that PDK1 binds membrane PIP3, then PDK1, phosphorylates and activates Akt. Akt is activated and stimulates several factors. Akt directly or indirectly regulates cell growth and cell survival through phosphorylating its substrates. The PI3K negative regulation and Akt deactivation are done by PTEN. PTEN is a phosphatase that removes phosphates group from the phosphatidylinositole 3, 4,5-3phosphate that causes Akt inactivation [62]–[64].

Some transcription factors are called STATs (signal transducers and activators of transcription) that perform as downstream effectors for signaling of cytokine and growth factor receptor. STAT proteins (STAT3 herein), is capable of binding directly to EGFR through its SH2 domain and is activated after binding. After being activated by tyrosine phosphorylation via its receptor, STAT3 becomes a dimmer [65], [66]. The activated STAT3 dimer moves to the nucleus and triggers expression of some genes necessary for proliferation and cell survival [67].

Based on literature survey, EGFR signaling network was modified for NSCLC and an expanded and reconstructed network was designed for the NSCLC cancerous state and it was dubbed NSCLC model which is accessible in table S2. Several studies have shown that the EGFR expression level increase is very common in the NSCLCs [16], [68], [69] and the EGFR concentration has been compared within several wild and cancerous lung cells [12], [13]. EGFR mutations in kinase domain are generally known as activating mutations because they seem to set off augmented kinase activity of the receptor [24]–[26]. In our study, we preceded to the L585R usual mutation dynamics which occur in exon 21 which belongs to the drug sensitive group of mutations. It is evident that ligand induced EGFR phosphorylation kinetics between wild type and mutant EGFR are different [17], [30], [31]. For example wild type receptor is phosphorylated much faster than the mutant one [17]. Additionally it is evident that activation-kinetics of downstream factors such as ERK [17], Akt [44] and STAT3/5 [32], [44] are different as well. The effect of wtEGFR and ΔEGFR internalization rate change experiments on EGFR dependent internalization in wtEGFR and ΔEGFR has shown it up to 60 min and indicated that wtEGFR internalization rate is roughly two times that of ΔEGFR [48]. PI3K, Akt, STAT3 and Ras increased nearly more than two folds on the basis of microarray expression data [70]. Cetin et al. reported PTEN gene expression reduction in 39% of NSCLC cases and in 26% of those which showed EGFR over expression [44].

### Computational Model

Systems biology involves network like aspects of biological systems so it’s not at a discrete molecular level. The scope of systems biology could be from an individual reaction cycle of a cell, tissue or even the whole organism. Mathematical modeling has a pivotal role in converting biological events into quantitative data for analysis. For better results, such models ought to reflect the systems as they really exist because by only this way, reliable predictions are feasible about them. - knowing that a model basically represents the topology of its components including the corresponding links, however, representation of the dynamic nature of the biological system provides the model with predictive power.

One of the most usually applied methods in order to model biological systems is ODE (Ordinary Differential Equations). A differential equation is defined as an equation showing the relationship between a function and some of the derivatives of that function. Principally a differential equation designates how a variable such as [S], i.e. the concentration of S, changes over the time. This is done by interrelating the rate of change with the concentration at the moment [71]–[74].

As an example, suppose the following reaction in which S is converted into P:
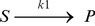
(1)


This is a simple reaction with no catalyst which can be modeled using Mass Action Kinetics [75], [76]. k denotes the rate constant of the reaction. Thus, the reaction goes on as below:
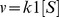



It is evident that the reaction rate (v) is directly related to [S], namely, the more the concentration of the S, the higher the reaction rate, hence the faster S is consumed the faster P is produced. Regarding the above equation, it would be easy to pose differential equations defining the rate of change in [S]and [P]:
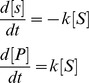



For modeling and simulating reaction 1, it is necessary to know S and P initial values. The reaction above is called a first-order reaction. In such reactions, the rate is relational to the value of a single reactant. On the other hand a second-order reaction is relational to the square of the value of an individual reactant or two reactants:
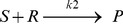
(2)


Where the reaction rate is:
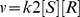
k1 and k2 are defined in S^−1^ and µM^−1^S^−1^ dimensions respectively.

According to what mentioned above, consider the enzymatic reaction below:
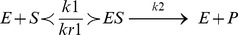



Primarily, enzymes (E) and substrates (S) bind together to form a complex (ES). The enzyme simplifies circumventing activation barrier, by which it accelerates the chemical change of the substrate into the product (P). Afterwards enzymes and products are split to form E and P. ES formation is described by a second order (in units of µM^−1^ s^−1^) forward rate constant (k1), a reversal rate constant (kr1; in s^−1^) of the first order, and also a first order catalytic rate constant (k2; in s^−1^).

The above enzymatic reaction rate, according to Michaelis-Menten [76], [77]equation is:
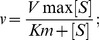



Here, [S] presents the concentration of substrate, Vmax is the maximum rate. The Michaelis constant km is the substrate concentration at which the reaction rate is half of the Vmax.
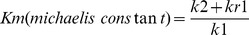



Vmax = k2 [E_t_], Et (the total amount of enzyme) =  [E] + [ES], Where k2 is rate limiting and as below:
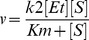



Our proposed normal model is based on ODEs and involves 109 species, 117 reactions, 187 parameters and 1 rule. Our proposed NSCLC model is based on ODEs and involves 109 species, 117 reactions, 188 parameters and 1 rule. In table S1 and table S2, reactions are shown in normal and NSCLC cells respectively. The tables also indicate the initial values for non-zero species in normal and cancerous cells respectively. The systems biology markup language (SBML) of our models is also provided in SBML S1 (normal model) and SBML S2 (normal model). SBML is a computer-readable format like XML for representing models of biochemical reaction networks. For simulations, ODE15s routine from MATLAB 7.9.0 was used to solve ODEs.

Here, a sample derivation regarding one of the ODEs (related to EGF and EGFR binding) is presented; the reaction is considered as a second order one.
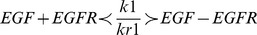



The reaction rate by which EGF-EGFR is produced is:
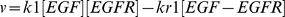



Where *k1* is the rate constant for the forward direction and *kr1* is for the reverse one.

## Results and Discussion

**Figure 2:**
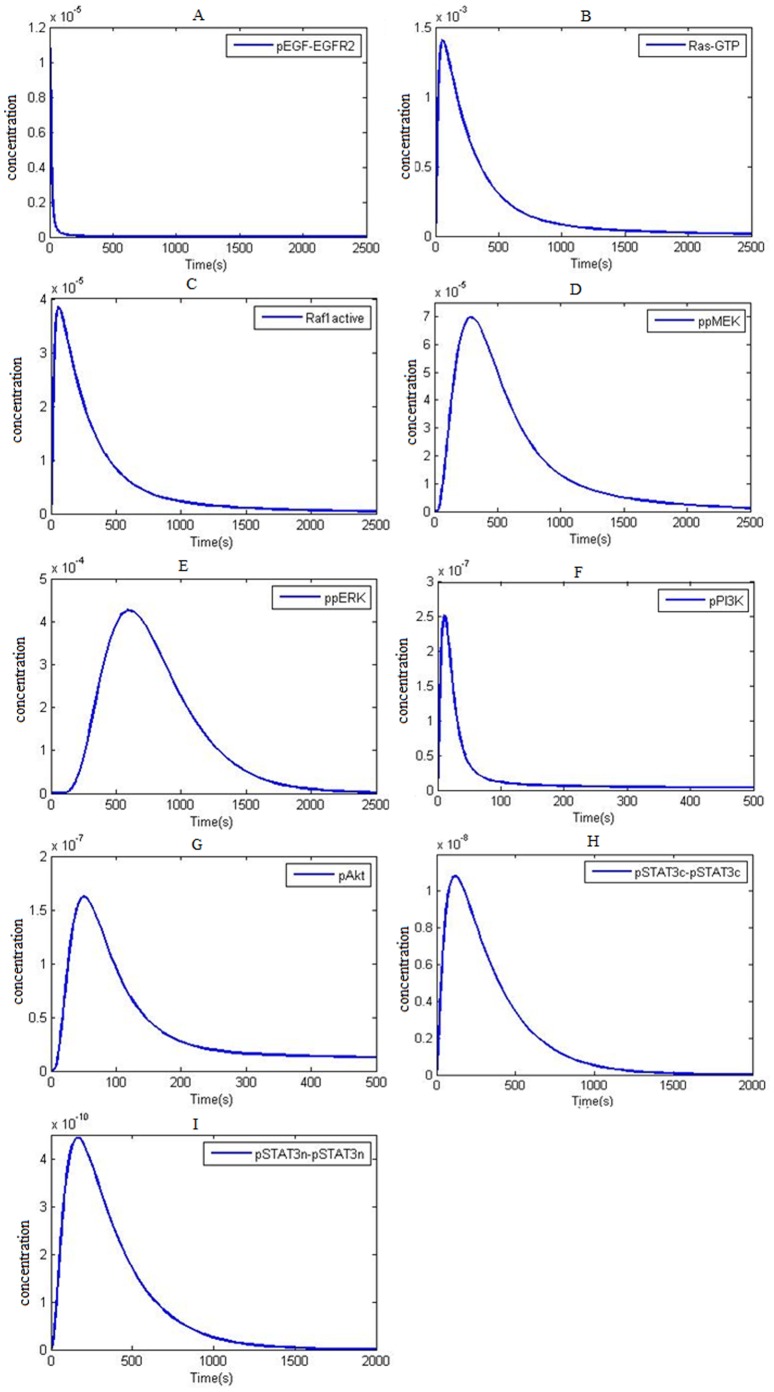
Normal EGFR signaling computational simulation at 50 ng/ml of EGF. A) Kinetics of EGFR autophosphorylation (pEGF-EGFR2). B) Kinetics of Ras-GTP formation. C) Kinetics of Raf1 activation (Raf1active). D) Phosphorylation kinetics of MEK leading to ppMEK double phosphorylation. E) Phosphorylation kinetics of ERK leading to ppERK double phosphorylation. f) Kinetics of PI3K phosphorylation (pPI3K). G) Activation kinetics of Akt as result of phosphorylation (pAkt). H) Kinetics of phosphorylated STAT3 dimerization in cytoplasm. I) kinetics of dimer STAT3 import into the nucleus.

Figure 2 shows the important species behavior in basic EGFR signaling pathways in normal cells that are modeled in accordance with table S1. The model calculates the rate of concentration fluctuations in 109 species by stimulation of EGF 50 ng/ml. Important species kinetics are shown in figure 2. In EGF 50 ng/ml, phosphorylation and activation peaks are depicted for important species in three pathways of Ras/ERK, PI3K/Akt and STAT activation. Figure 2 shows EGFR phosphorylation kinetics (figure 2A), Ras-GTP formation kinetics (figure 2B), Raf-1 activation kinetics (figure 2C), MEK phosphorylation kinetics (figure 2D) and ERK or ERK phosphorylation kinetics (figure 2E). From PI3K/Akt pathway, phosphorylation and activation kinetics of two important factors PI3K (figure 2F) and Akt (figure 2G) and from the STAT activation pathway, Phosphorylated STAT3 dimerization kinetics in cytoplasm (figure 2H) and import STAT3 dimmer from the cytoplasm to the nucleus (figure 2I) are shown. Activation of ERK that is a causative of cell proliferation and ERK phosphorylation peak was predicted at the 10^th^ minute and as manifested by figure 2E the level of ERK is highly reduced within 2000 seconds. Akt which is the main agent of cell survival shows the phosphorylation peak at about 50 seconds (figure 2G). The highest dimer STAT3 concentration is at the 200^th^ second. Its concentration reduces with a mild slope over the time (figure 2H). STAT3 plays an important role in cell proliferation and survival.

### EGFR Over Expression and Mutated EGFR Effect

There are many reports on EGFR over expression in the NSCLC. Tsakiridis et al. have stated the presence of the activated membrane EGFR in NSCLC [78]. Hirish et al. showed that the over expression occurred in 62% of NSCLCs [16]. Mukohara et al. identified EGFR over expression in 78% of NSCLCs [79]. Rush et al. reported EGFR over expression in 98% of NSCLs by means of Northern analysis [68]. The pulmonary carcinogenic and membrane EGFR concentrations were compared with a number of reports. The mean EGFR concentration in tumors was 338 fmolmg^−1^, in normal lung cells it was 10.26 fmolmg^−1^ and this concentration in stage IV of cancer is more than stage I and II in a meaningful way. The normal/pathogenic cut-off point is considered as 12.9 fmolmg^−1^ [12]. Dittadi has also approved this cut-off but EGFR concentration in normal lung tissue was 7.4 fmolmg^−1^ and in cancerous conditions it was 23.5 fmolmg^−1^ [13]. Veale et al. proposed a cut-off for the death risk and mentioned that if EGFR concentration is more than 35 fmolmg^−1^ in plasma membrane, the death risk would increase [80]. The results demonstrate the pivotal role of the EGFR gene copy number increase regarding EGFR over expression. Hirsh et al. via FISH [16], Gandi et al. via qPCR, CGH and FISH [81] showed a significant correlation between EGFR gene copy number increase with increasing EGFR expression. Reinmuths findings are in accordance with this fact [82]. We also set the EGFR concentration as three times the normal model in NSCLC model.

**Figure 3:**
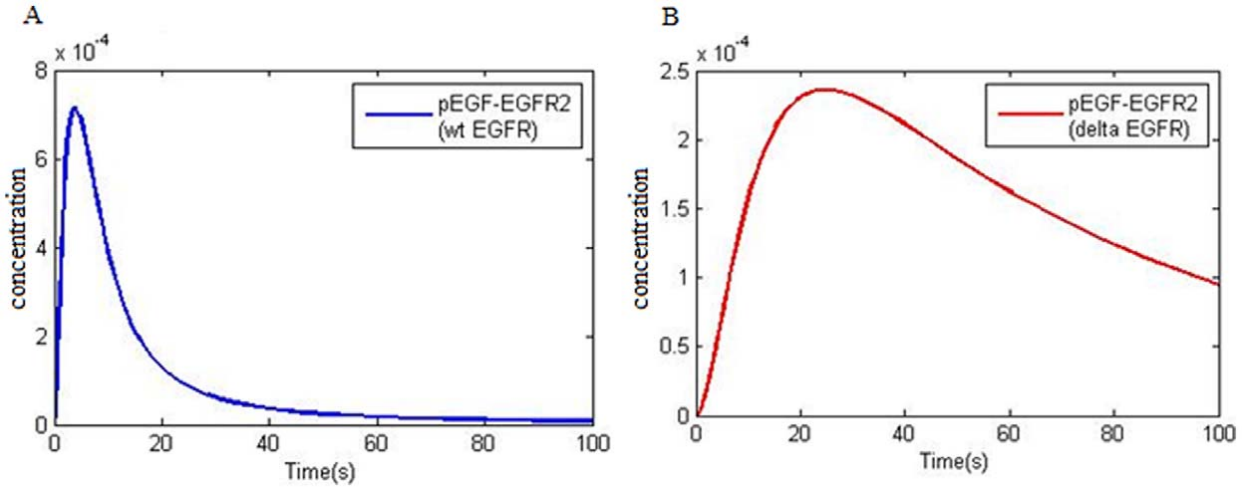
Computational simulation of EGFR and ΔEGFR autophosphorylation and internalization at 50 ng/ml EGF. A) Kinetics of EGFR autophosphorylation and internalization in 100secs. B) Kinetics of Δ EGFR autophosphorylation and internalization in 100 secs.

The studies on HCC827 cells having gene amplification and exon 19 deletion in EGFR gene and H1819 cells showing over expression of ERBB was done by Amann et al. The mentioned studies showed that EGFR mutation regarding EGFR gene amplification happens in NSCLC cells [17]. In several reports the kinetic difference between mutated EGFR (ΔEGFR) and wild type EGFR has been discussed. Simulation results showed that wtEGFR and ΔEGFR (L858R) phosphorylation kinetics are different from one another (figure 3) and shows that EGFR phosphorylation peak in ΔEGFR in comparison with wtEGFR is delayed. The kinetics of ΔEGFR and wtEGFR were reported to be different disclosing that the EGFR phosphorylation peak in ΔEGFR was accompanied with latency compared with wtEGFR [17]. Ashi et al. also reported that EGFR phosphorylation peak in ΔEGFR in comparison with wtEGFR is lagging. Several studies in this case have shown that phosphorylation kinetics of wtEGFR and ΔEGFR are different [83]–[86].

Our simulation results also demonstrate the difference between EGF dependent internalization rate of ΔEGFR and wtEGFR receptors. Figure 3 illustrates the different kinetics of EGF dependent internalization rates for ΔEGFR and wtEGFR. The results showed that in 50 ng/ml EGF, the amount of normal cell surface receptor (wtEGFR) was rapidly decreasing but in ΔGFR (NSCLC) it decreased with a mild slope.

The experiment demonstrated that wtEGFR and ΔEGFR internalization rates are different. Cell surface wtEGFR level halved proportional to ΔEGFR in a way that after 120 min, cell surface’s wtEGFRs would vanish, conveying their internalization, however a considerable amount of ΔEGFR was remaining. This fact discloses that wtEGFR internalization rate is roughly two times that of ΔEGFR [87].

In another experiment it has been shown that after the addition of EGF, ΔEGFRs remain on the cell surface for a while, but wtEGFRs are wiped from the cell surface [88], [89], since rate-limiting step in down regulation is receptor internalization from the cell surface, therefore the endocytosis rate calculation is important [88], [90]. Our simulation results verify that internalization plays an important role in down regulation. If in NSCLC model (table S2) internalization rate parameters are constant, even through EGFR concentration and ΔEGFR related parameter changes, downstream factor kinetics will roughly remain constant which indicates that the activity levels of ERK, STAT3 and Akt are approximately the same level related to the normal state (data not shown).

### Comparison of Ras/ERK Pathways between Normal and NSCLC Model

**Figure 4:**
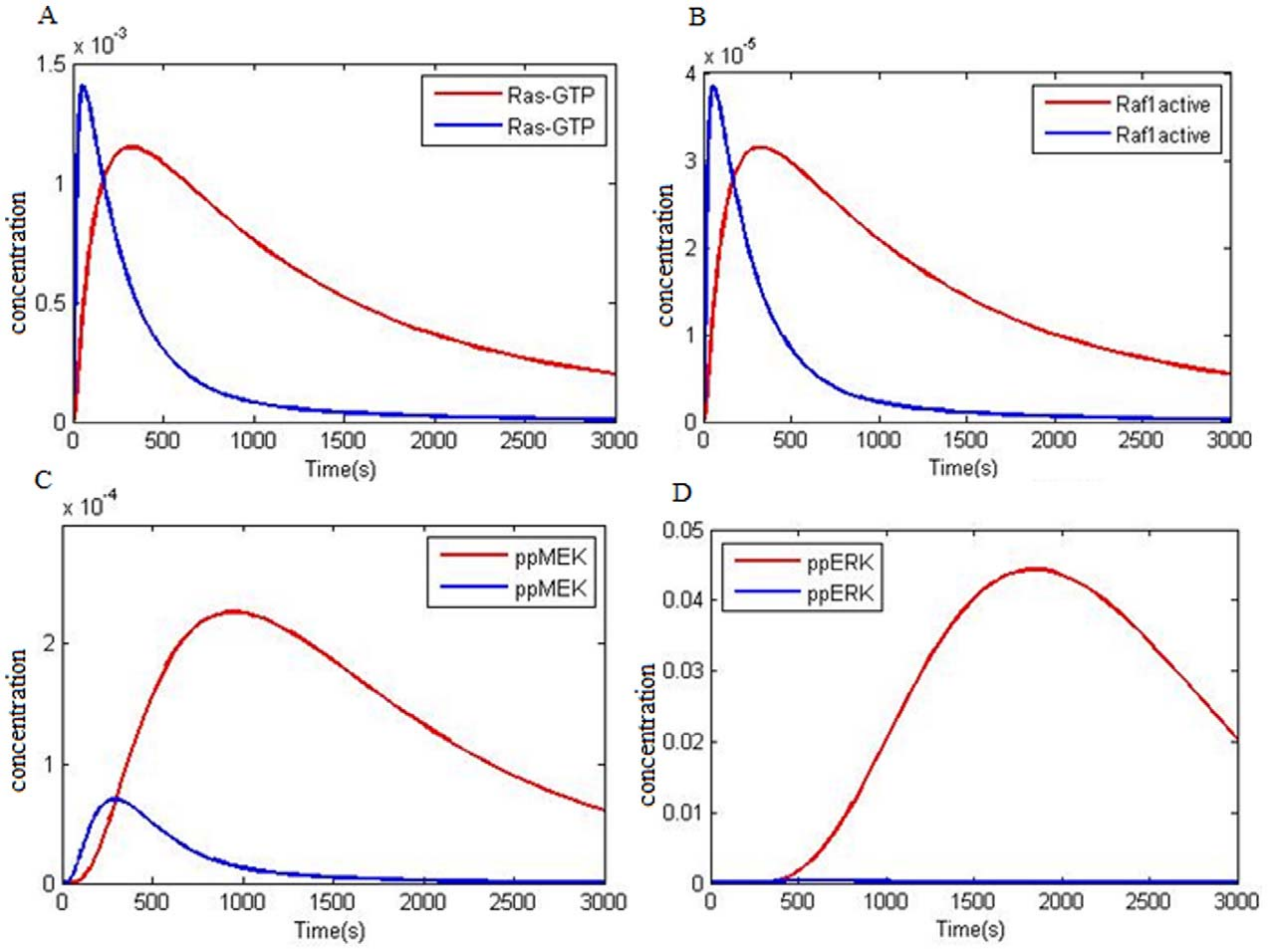
Important species kinetics comparison in Ras-ERK pathway between normal and NSCLC models at 50 ng/ml EGF. A) Kinetics of Ras-GTP formation. B) Kinetics of Raf1 activation. C) Phosphorylation kinetics of MEK leading to ppMEK double phosphorylation. D) Phosphorylation kinetics of ERK leading to ppERK double phosphorylation: **NSCLC model factor (red); normal model factors (blue).**

Activated ERK (phosphorylated(P)-ERK) has been extracted from 84% of 60 prostate cancer samples, 91% out of 101 head and neck cancer samples and 67% out of 74 gastric cancer samples and 72% out of 90 breast cancer samples, respectively [91], [92]. Also Oka et al. [93] and Ito et al. [94], have reported P-ERK in 48% of renal and 58% of hepatocellular carcinoma cancerous samples, respectively. Mukohara has revealed that ERK activation is related to EGFR signaling in NSCLC [79]. Vicent et al. evaluated 111 NSCLC samples and 30 normal lung tissues and reported that ERK1/2 (P-ERK) activity in 30 normal lung tissues were negative and 34% of NSCLCs showed active ERK1,2 [95]. Amann et al. reported that wtEGFR and ΔEGFR kinetics are different and activated ERK via ΔEGFR sustained its activity for a longer time. On the other hand two mutations in EGFR (del L747-P753, L858R) in two different kinetic studies with wtEGFR showed that above mutations affect the downstream pathways. These downstream pathways are particularly related to anti apoptotic pathways i.e. STAT3/5 and PI3K/Akt activation and denote low effect of EGFR mutations on ERK activation [24], [96], but, boactivities studies have demonstrated ERK1,2 activity in NSCLC [17], [79], [95]. Our simulation results showed that mutations in EGFR (L858R), enhanced EGFR and Ras expression in NSLC had a deep impact on Ras-ERK. Figure 4 compares important species kinetics in Ras/ERK between normal model and NSCLC and demonstrates that Ras/ERK pathway activation kinetics is vastly different between the normal model and NSCLC. Figure 4A compared Ras-GTP formation kinetics. Ras-GTP formation peak in NSCLC (red line) is lagging relative to the normal one, but its concentration decreases with a mild slope. Raf1 activation kinetics (Raf1active) are also similar to Ras-GTP formation (figure 4B). MEK downstream factor kinetics and ERK between the normal and NSCLC model are completely different. Figure 4C shows the MEK phosphorylation peak in NSCLC (red line) in about 1000 seconds while in the normal model it is about 500 seconds. Phosphorylated MEK (ppMEK) in NSCLC is much higher than the normal model indicating that in NSCLC MEK activity is higher and the active state period is longer for MEK. Figure 4D shows that activity level or phosphorylation of ERK (ppERK) in NSCLC (red line) is not comparable to the normal cell in a way that conversely, normal model ppERK concentration is trace. Normal model ERK phosphorylation kinetics in figure 2E shows that phosphorylation peak occurs within 600 seconds but the simulation predicts that NSCLC ERK phosphorylation peak is nearly 2000 seconds. Also, the concentration and the time period of phosphorylated ERK in NSCLC is much more that the normal model.

### Comparison of PI3K/Akt and STAT Activation Pathway between Normal and Cancer Cell

**Figure 5:**
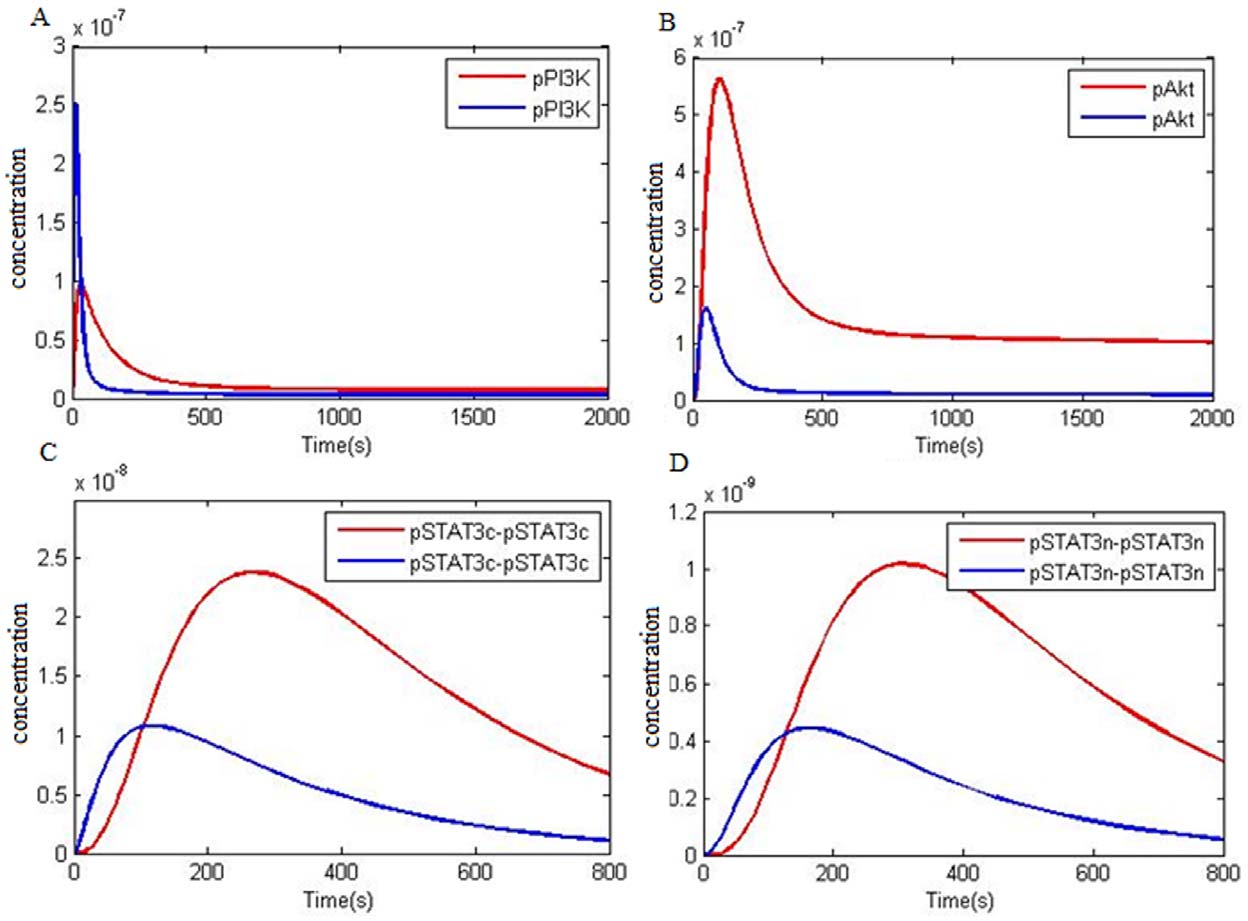
Important species kinetics comparison in PI3K/Akt and STAT3 activation pathways between normal and NSCLC models at 50 ng/ml EGF. A) Kinetics of PI3K phosphorylation. B) Activation kinetics of Akt as result of phosphorylation. C) Kinetics of phosphorylated STAT3 dimerization in cytoplasm. D) Kinetics of dimer STAT3 import into the nucleus: **NSCLC model factor (red); normal model factors (blue).**

Shigematsu et al. [96] and Sordella et al. [24] disclosed the activity of PI3K/Akt pathway and activity of STAT 3/5 in NSCLC. P-STAT3 has been observed in 46% of lung adenocarcinoma cases and it has been reported that P-EGFR is commonly expressed in P-STAT3 [97]. Pong demonstrated that reducing STAT3 expression reduces the apoptotic rate in NSCLC and proposed that STAT3 over expression is related to NSCLC cell growth and survival [98]. Studies have proved a strong correlation between P-EGFR, P-STAT3 [32], [99], P-Akt [100] and P-ERK [79] in NSCLC [100]. Ganti examined the EGFR behavior in different malignancies such as NSCLC and pinpointed its role in triggering several factors such as PI3K and STAT3/5 [101]. Studies by Mukohara et al. showed that P-ERK, P-Akt and P-STAT was detected in 28%, 53% and 58% of NSCLCs respectively while half the samples showed simultaneous expression of 2 or 3 mentioned factors indicating various signal transduction routes in NSCLC [79]. Figure 5A and Figure 5B compares PI3K and Akt activation kinetics between NSCLC and the normal model. In figure 5B, it is pointed that Akt (pAkt) activity level in NSCLC (red line) is higher than the normal model, and by stimulating 50 ng/ml EGF after the peak, its activity level is sustained for a long time. These instances show that mutations in EGFR and enhanced levels of PI3K and EGFR expression have a considerable effect on PI3K pathway.

Figure 5C shows that formation of cytoplasmic STAT3 dimer (pSTAT3c-pSTAT3c) in NSCLC (red line), has different kinetics compared to the normal model and the concentration level of pSTAT3C-pSTAT3C is much higher than the normal cell. Also, it remains in the cell for a longer period of time so that after 800 seconds the level is still high. It is determined in the figure 5C that dimer STAT3 formation peak in NSCLC as compared to the normal model is lagging. Figure 5D shows the kinetics behavior of nuclear dimer STAT3 (pSTAT3n-pSTAT3n). Nuclear dimer STAT3 in NSCLC (red line) has a higher level of concentration compared with the normal model and remains in the nucleus for a longer time in a way that after 800 seconds its level is still high. These results indicate that EGFR mutation, STAT3 and EGFR over expression deeply affect STAT activation pathway.

### Sensitivity of NSCLC Cells to TKI (Tyrosine Kinase Inhibitors)

**Figure 6:**
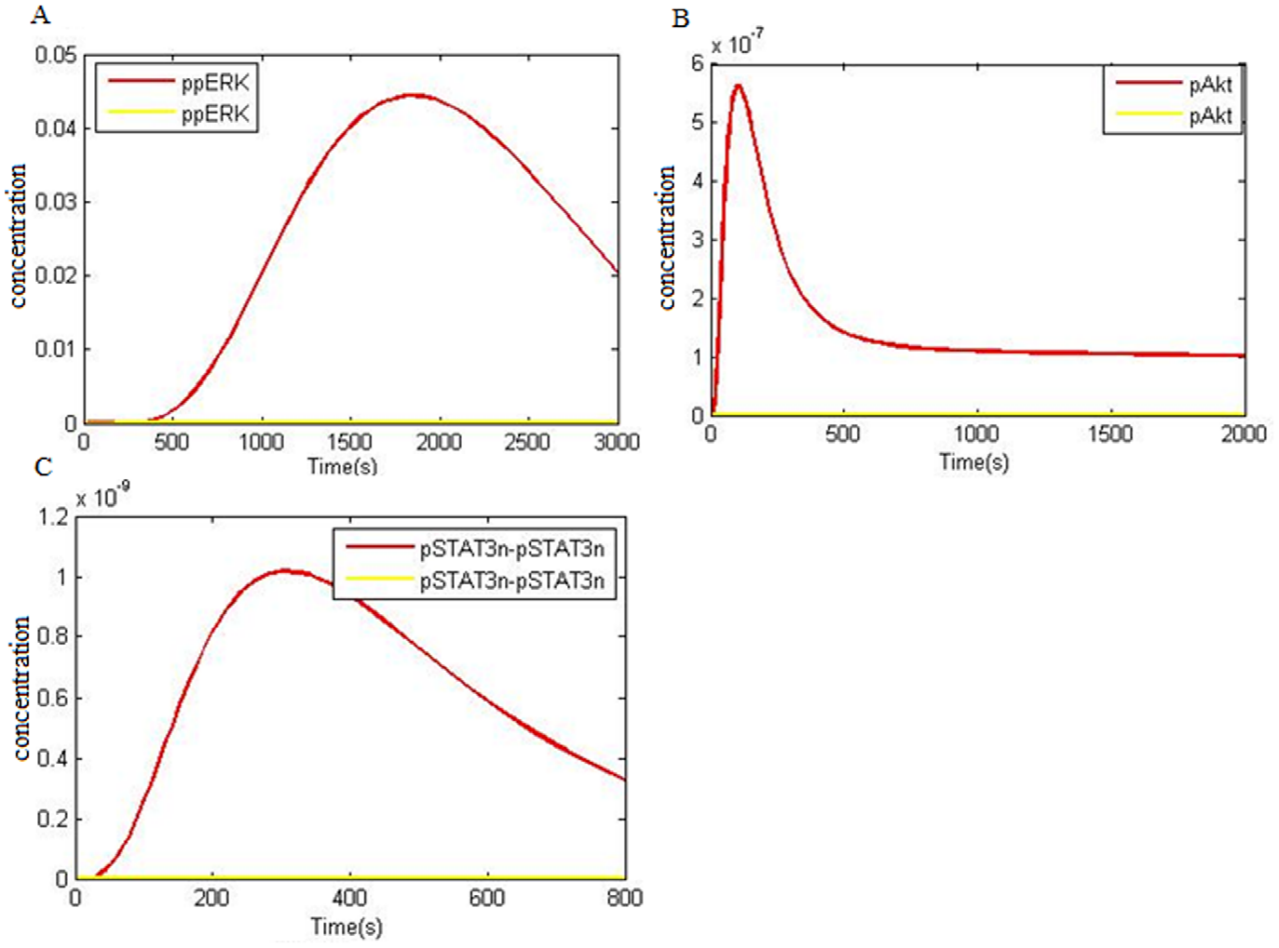
Inhibition of EGFR signaling in the presence of erlotinib (IC 50 ) and comparison with kinetics of three important factors in NSCLC model (at 50 ng/ml EGF). A) Phosphorylation kinetics of ERK leading to ppERK double phosphorylation. B) Activation kinetics of Akt as result of phosphorylation. C) Kinetics of dimer STAT3 localization into the nucleus: **NSCLC model factors (red); NSCLC model factors in the presence of erlotinib (yellow).**

Regarding corresponding findings, TKIs (gefitinib and erlotinib) are effective against NSCLC tumors containing EGFR gene mutations [17], [25], [31], [32], [84], [100], [102]. H1819 cells showed to be moderately sensitive (IC50 4.7 micromol/L) and HCC827 cells turned out to be highly sensitive (IC50 10 nmol/L) to gefitinib. Similar to gefitinib, erlotinib also inhibited the growth of H1819 and HCC827 cell lines (IC50 5.0 and 0.010 micromol/L, respectively) but did not affect the H1299 cells (IC50 50.0 micromol/L). Amann et al. reported that IC50 of TKIs showed a more closely correlation with the inhibition of ERK and Akt phosphorylation and also pointed that inhibition of EGFR tyrosine kinase activity by means of gefitinib inhibits downstream STAT3 activity [17]. In H3255 cells with L858R mutation, Haura et al. found that EGFR tyrosine kinase inhibition by TKI leads to complete inhibition of pSTAT3 [32]. This implies that loss of STAT3 activity triggers gefitinib mediated apoptosis. Sordella et al. demonstrated that cancerous Lung cells containing ΔEGFR were more sensitive to gefitinib as much as 100-fold higher than cells with wtEGFR. It was also shown that gefitinib inhibits PI3K, Akt and STAT activation pathways through effect on EGFR [24]. Li et al. [103] and Okamoto et al. [104] reported that apoptosis and inhibition of proliferation in response tpathway because of PI3K/Akt and Ras/ERK pathways inhibition. Our simulation shows that the activity of final factors of three downstream pathways i.e. ERK, Akt and nuclear dimer STAT3 in 10 µmol/liter erlotinib (IC50) is inhibited. Figure 6 shows the downstream factor activity inhibition in the presence of erlotinib which compare with their active state in NSCLC at EGF 50 ng/ml concentration. The results comply with Emery et al. [100] who reported that TKI leads to decrease of pAkt, pSTAT3 and pERK levels.

### Loss of PTEN Expression Effect on PI3K/Akt Pathway in NSCLC Model

**Figure 7:**
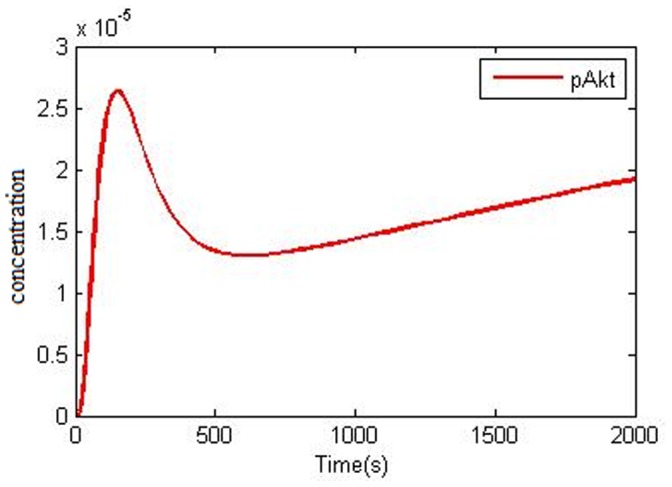
Computational simulation of Akt phosphorylation (pAkt) in NSCLC that involve loss of PTEN (at 50 ng/ml EGF). For more information refer to the context.

PTEN is responsible for PI3K/Akt pathway negative regulation and Akt deactivation. PTEN is a phosphatase that removes phosphates group from the phosphatidylinositole 3,4,5-3phosphate that causes Akt inactivation [62]–[64]. Loss of PTEN (as a key protein in EGFR signaling) is common in NSCLCs [105], [106]. Cetin et al. reported PTEN gene expression reduction in 39% of NSCLC cases and in 26% of those which showed EGFR over expression [44]. Marsit et al. [107] and Soria et al. [108] also proposed that there is no correlation between PTEN expression level and tumor properties in NSCLC. However Tang et al. [105] and Lim et al. [109] proposed that loss of PTEN expression on NSCLC is related to Lymph node distant metastasis and also later stages. Cetin et al. experiment showed that low PTEN expression might be related to low survival rates in NSCLC patients [44]. Our simulation shows that (figure 7) by PTEN loss (see SBML S3) i.e. when PIP3 is not changed into PIP2, negative control of PI3K/Akt pathway is removed and activity level of pAkt in NSCLC is highly increased. These results show the importance of PTEN in negative control of the PI3K/Akt. In figure 7, it is clear that after Akt phosphorylation peak, the level of Akt is slightly decreased, but after a period of time it increases with a mild slope so that after 2000 secs the pAkt is at its highest level. This pAkt level as compared to “NSCLC model with normal PTEN” pAkt (figure 5B) level is high and after some time its level goes even higher.

### Loss of PTEN Expression and Intrinsic Resistance to TKI

**Figure 8:**
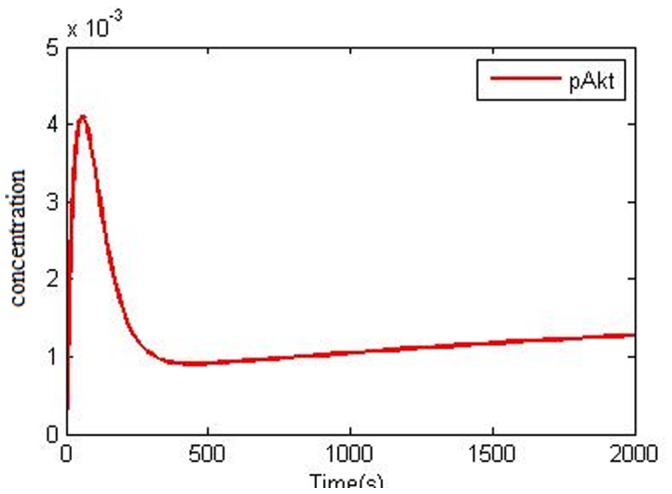
Computational simulation of Akt phosphrylation (pAkt) in 10 µmol/liter erlotinib (IC 50) in NSCLC that involve loss of PTEN (at 50 ng/ml EGF).

Several studies have introduced PTEN expression reduction as the instinctive resistance factor against TKI. Kokubo et al. showed that PTEN expression stops adenocarcinoma progression because resistance to gefitinib is lost [109]. Endoh et al. evaluated the relationship between patient’s survival and PTEN mRNA expression levels in Gefitinib cleft in mutated EGFR containing NSCLC and proved that survival was longer in high PTEN expression compared with low PTEN expression [110]. Cetin et al. proved that loss of PTEN expression could work as an intrinsic strategy for EGFR tyrosin kinase resistance in NSCLC [44]. Several studies have highlighted the lack of PTEN expression in cases of being resistant to erlotinib because of NSCLC with EGFR mutation [112]–[113]. If PTEN is mutated, PIP3 level would not decrease in the cell, therefore in the presence of erlotinib, even though upstream factors are inhibited, the level of pAkt remains constant as a consequence of PTEN loss (figure 8). Therefore cells show resistance to erlitinib due to a constant pAkt level which is one of the pivotal factors for cell survival.

### Conclusion

In this study, we have built mathematical models representing EGFR signaling in normal and non-small cell lung cancer (NSCLC) EGFR signaling, and explore the different dynamics and bevariors of these models. For the first time we simultaneously analyze the mutation in both EGFR and PTEN and over-expression of PI3K, EGFR, Akt, STAT3 and Ras in NSCLC EGFR signaling in one study. Various studies have shown that EGFR signaling plays a very important role in NSCLCs [6], [7], [16]–[22], [68], [69], [78]–[81]. Since, EGFR signaling is crucial for cell survival and proliferation; it might be the main reason for tumor progression in NSCLC. EGFR signaling activates Ras/ERK, PI3K/Akt and STAT activation pathways. These three pathways are the main routes for cell proliferation and survival [1], [4], [5], [57]–[59], [62]–[67]. Therefore mutations that lead to excessive activation of these pathways may cause cancer. One of these mutations is an EGFR mutation which is frequently seen in NSCLC samples. This mutation causes kinetic changes in downstream factors in the above pathways. The result of this mutation is tumorgenesis and tumor progression. Activation mutations in kinase domain which is often accompanied with EGFR expression, can change receptor phosphorylation kinetics in wtEGFR and ΔEGFR (herein L858R) [17], [83]–[86]. Figure 3, illustrates that phosphorylation pattern and phosphorylation peak are different between wtEGFR and ΔEGFR, whilst, phosphorylation peak is delayed in ΔEGFR.

Moreover, figure 3 shows that internalization rate is different between ΔEGFR and wtEGFR in a way that reduction in phosphorylated cell surface ΔEGFR occurs with a mild slope so that after 100 seconds a considerable level of phosphorylated ΔEGFR is observed. Accordingly, reduction level of the membrane receptor (i.e. EGF dependent internalization rate) after a while is slower than that of wtEGFR. These results are consistent with some reports in this field [87]–[89]. We predicted that the rate limiting step in downstream is cell surface ΔEGFR internalization. By comparing two models in the case of constant internalization rate parameters in NSCLC model, even by changing other kinetic factors, no considerable change in downstream factors happens (data not shown).

Microarray data [70] shows that some downstream factor expression levels such as Akt, PI3K, STAT3 and Ras in NSCLC is roughly doubled. In addition, the above mentioned items related to EGFR are also effective on downstream factor kinetics. Our simulation denotes the effect of EGFR mutations and increased expression of certain factors in NSCLC EGFR signaling on each of the three pathways namely Ras/ERK (figure 4), PI3K/Akt (figure 5 A,B) and STAT activation (figure 6C,D). Some reports demonstrated high level of pSTAT in NSCLC with EGFR mutation [32], [97]–[99]. Four arrays of reports have revealed an increase in PI3K/Akt and pSTAT3 in NSCLC with EGFR mutation [24], [96], [100], [101]. Shigematsu et al. [96] and Sordella et al. [24] believe that EGFR mutation is specially exerting effects on PI3K/Akt and STAT3 having a minute effect on ERK activation (Ras/ERK pathway). But Mukohara et al. [79] have shown that in NSCLC samples, frequency levels of all three pSTAT3, pAkt and pERK are high and in half the samples, high levels of two or three factors are observed simultaneously. Besides, Amann et al. [17] and Vicent et al. [95] have shown that in NSCLC samples with EGFR mutation, pERK level is high. Our simulation shows that mutations in EGFR exerts effect on all three pathways so that pERK, pSTAT and pAkt levels are increased. Over activation of ERK, Akt and STAT3 which are the main cell proliferation and survival factors is a promoting factor for tumor progression in NSCLCs.

Loss of PTEN expression is common in NSCLC samples [44], [105], [106] and knowing that PTEN is an important protein in negative control of PI3K/Akt pathway [62]–[64], in case of loss of PTEN, Akt activity level is drastically increased (figure 7) that compared with NSCLC sample without loss of PTEN (figure 5B) Akt level was also increased.

Various reports have evaluated TKI effects on NSCLC with EGFR mutation [17], [25], [31], [32], [84], [100], [102]. TKI causes apoptosis and causes cell proliferation inhibition in NSCLC with EGFR mutation. The inhibition of PI3K/Akt, STAT3 activation and Ras/ERK pathways leads to apoptosis and cell proliferation inhibition, the result of which is tumor progression inhibition. Our simulation results show that in 10 µmol/liter erlotinib (IC50), downstream factors pAkt, pSTAT3 and pERK are inhibited by comparison with NSCLC model in figure 6. However, in case of loss of PTEN expression in the presence of erlotinib, pAkt level would not decrease (Figure 8) and as pAkt level does not decrease, these cells are resistant to erlotinib. Our prediction is in compliance with studies of Cetin et al. [44] and Endoh et al. [110].

## Supporting Information

Table S1 **Normal cell reactions and parameters.**
(DOCX)

Table S2 **NSCLC reactions and parameters.**
(DOCX)

SBML S1 **SBML file for normal cell.**
(XML)

SBML S2 **SBML file for NSCLC.**
(XML)

SBML S3 **SBML file for NSCLC** **(loss of PTEN).**
(XML)
