# Supplementary material for: Modeling of Tumor Progression in NSCLC and Intrinsic Resistance to TKI in Loss of PTEN Expression
Source: PLoS One. 2012 Oct 24;7(10):e48004. doi: 10.1371/journal.pone.0048004 (PMC3483873; doi:10.1371/journal.pone.0048004)
Supplement: Table S1 — Normal cell reactions and parameters. (DOCX) [file pone.0048004.s001.docx]

**Supplementary Table 1**

1. **Normal model reactions**

Note: Michaelis Menten constants are given in µM, first order rate constants in s^-1^ and second order rate constants in µM^-1^ s^-1^

| **reactions** | | **parameters** | | **References** |
| --- | --- | --- | --- | --- |
| **r1** | **EGF + EGFR <-> [EGF-EGFR]** | **K1= 100** | **Kr1= 0.0038** | **(**[**Schoeberl et al, 2002**](#_ENREF_80)**;** [**Yamada et al, 2004**](#_ENREF_102)**)** |
| **r2** | **[EGF-EGFR] + [EGF-EGFR] <-> [EGF-EGFR2]** | **K2= 10** | **Kr2= 0.02** | **(**[**Sasagawa et al, 2005**](#_ENREF_79)**;** [**Yamada et al, 2004**](#_ENREF_102)**)** |
| **r3** | **[EGF-EGFR2] -> [pEGF-EGFR2]** | **K3=2.014** |  | **(**[**Yamada et al, 2004**](#_ENREF_102)**)** |
| **r6** | **[pEGF-EGFR2] + SHP2 <-> [pEGF-EGFR2-SHP2]** | **K6=3.114** | **Kr6=0.2** | **(**[**Yamada et al, 2004**](#_ENREF_102)**)** |
| **r7** | **[pEGF-EGFR2-SHP2] -> [EGF-EGFR2] + SHP2** | **K7=2.661** |  | **(**[**Yamada et al, 2004**](#_ENREF_102)**)** |
| **r8** | **[pEGF-EGFR2] + Shc <-> [pEGF-EGFR2-Shc]** | **K8=90** | **Kr8=0.6** | **(**[**Yamada et al, 2004**](#_ENREF_102)**)** |
| **r9** | **[pEGF-EGFR2-Shc] -> [pEGF-EGFR2-pShc]** | **K9=0.5838** |  | **(**[**Yamada et al, 2004**](#_ENREF_102)**)** |
| **r10** | **[pEGF-EGFR2-pShc] <-> [pEGF-EGFR2] + pShc** | **K10=4.481** | **Kr10=0.3** | **(**[**Yamada et al, 2004**](#_ENREF_102)**)** |
| **r11** | **pShc + SHP2 <-> [pShc-SHP2]** | **K11=3.114** | **Kr11=0.2** | **(**[**Yamada et al, 2004**](#_ENREF_102)**)** |
| **r12** | **[pShc-SHP2] -> Shc + SHP2** | **K12=0.2661** |  | **(**[**Yamada et al, 2004**](#_ENREF_102)**)** |
| **r13** | **pShc -> Shc** | **K13=0.005** |  | **(**[**Yamada et al, 2004**](#_ENREF_102)**)** |
| **r18** | **[pEGF-EGFR2-pShc] + Grb2 <-> [pEGF-EGFR2-pShc-Grb2]** | **K18=3** | **Kr18=0.1** | **(**[**Kholodenko et al, 1999**](#_ENREF_38)**)** |
| **r19** | **[pEGF-EGFR2-pShc-Grb2] + SHP2 <-> [pEGF-EGFR2-pShc-Grb2-SHP2]** | **K19=10** | **Kr19=1** | **(**[**Yamada et al, 2004**](#_ENREF_102)**)** |
| **r20** | **[pEGF-EGFR2-pShc-Grb2-SHP2] -> [EGF-EGFR2] + pShc + Grb2 + SHP2** | **K20=2.661** |  | **(**[**Yamada et al, 2004**](#_ENREF_102)**)** |
| **r23** | **[pEGF-EGFR2-pShc-Grb2] + SOS <-> [pEGF-EGFR2-pShc-Grb2-SOS]** | **K23=10** | **Kr23=0.0214** | **(**[**Kholodenko et al, 1999**](#_ENREF_38)**)** |
| **r26** | **Grb2 + SOS <-> [Grb2-SOS]** | **K26=0.1** | **Kr26=0.0015** | **(**[**Kholodenko et al, 1999**](#_ENREF_38)**)** |
| **r27** | **[pEGF-EGFR2-pShc] + [Grb2-SOS] <-> [pEGF-EGFR2-pShc-Grb2-SOS]** | **K27=10** | **Kr27=0.045** | **(**[**Kholodenko et al, 1999**](#_ENREF_38)**)** |
| **r28** | **[pEGF-EGFR2] + Grb2 <-> [pEGF-EGFR2-Grb2]** | **K28=3** | **Kr28=0.05** | **(**[**Kholodenko et al, 1999**](#_ENREF_38)**)** |
| **r29** | **[pEGF-EGFR2-Grb2] + SHP2 <-> [pEGF-EGFR2-Grb2-SHP2]** | **K29=10** | **Kr29=1** | **(**[**Yamada et al, 2004**](#_ENREF_102)**)** |
| **r30** | **[pEGF-EGFR2-Grb2-SHP2] -> [EGF-EGFR2] + Grb2 + SHP2** | **K30=2.661** |  | **(**[**Yamada et al, 2004**](#_ENREF_102)**)** |
| **r31** | **[pEGF-EGFR2-Grb2] + SOS <-> [pEGF-EGFR2-Grb2-SOS]** | **K31=10** | **Kr31=0.06** | **(**[**Kholodenko et al, 1999**](#_ENREF_38)**)** |
| **r32** | **[pEGF-EGFR2] + [Grb2-SOS] <-> [pEGF-EGFR2-Grb2-SOS]** | **K32=2.734** | **Kr32=0.025** | **(**[**Yamada et al, 2004**](#_ENREF_102)**)** |
| **r35** | **[pEGF-EGFR2-pShc-Grb2-SOS] + [Ras-GDP] <-> [pEGF-EGFR2-pShc-Grb2-SOS-Ras-GDP]** | **K35=202.9** | **Kr35=0.18** | **(**[**Yamada et al, 2004**](#_ENREF_102)**)** |
| **r36** | **[pEGF-EGFR2-pShc-Grb2-SOS-Ras-GDP] -> [pEGF-EGFR2-pShc-Grb2-SOS] + [Ras-GTP]** | **K36=0.1434** |  | **(**[**Yamada et al, 2004**](#_ENREF_102)**)** |
| **r37** | **[pEGF-EGFR2-Grb2-SOS] + [Ras-GDP] <-> [pEGF-EGFR2-Grb2-SOS-Ras-GDP]** | **K37=202.9** | **Kr37=0.18** | **(**[**Yamada et al, 2004**](#_ENREF_102)**)** |
| **r38** | **[pEGF-EGFR2-Grb2-SOS-Ras-GDP] -> [pEGF-EGFR2-Grb2-SOS] + [Ras-GTP]** | **K38=0.1434** |  | **(**[**Yamada et al, 2004**](#_ENREF_102)**)** |
| **r39** | **[Ras-GTP] -> [Ras-GDP]** | **K39=0.000167** |  | **(**[**Yamada et al, 2004**](#_ENREF_102)**)** |
| **r40** | **[Ras-GTP] + [Ras-GAP] <-> [Ras-GTP-Ras-GAP]** | **K40=2.854** | **Kr40=0.96** | **(**[**Yamada et al, 2004**](#_ENREF_102)**)** |
| **r41** | **[Ras-GTP-Ras-GAP] -> [Ras-GDP] + [Ras-GAP]** | **K41=7.76** |  | **(**[**Yamada et al, 2004**](#_ENREF_102)**)** |
| **r42** | **[pEGF-EGFR2] + [Ras-GAP] <-> [pEGF-EGFR2-Ras-GAP]** | **K42=0.1** | **Kr42=0.01** | **(**[**Yamada et al, 2004**](#_ENREF_102)**)** |
| **r43** | **[pEGF-EGFR2-Ras-GAP] + [Ras-GTP] <-> [pEGF-EGFR2-Ras-GAP-Ras-GTP]** | **K43=2.845** | **Kr43=0.96** | **(**[**Yamada et al, 2004**](#_ENREF_102)**)** |
| **r44** | **[pEGF-EGFR2-Ras-GAP-Ras-GTP] -> [pEGF-EGFR2-Ras-GAP] + [Ras-GDP]** | **K44=7.76** |  | **(**[**Yamada et al, 2004**](#_ENREF_102)**)** |
| **r45** | **[pEGF-EGFR2-Ras-GAP] + SHP2 <-> [pEGF-EGFR2-Ras-GAP-SHP2]** | **K45=3.114** | **Kr45=0.2** | **(**[**Yamada et al, 2004**](#_ENREF_102)**)** |
| **r46** | **[pEGF-EGFR2-Ras-GAP-SHP2] -> [EGF-EGFR2] + [Ras-GAP] + SHP2** | **K46=2.661** |  | **(**[**Yamada et al, 2004**](#_ENREF_102)**)** |
| **r47** | **Raf1 + [Ras-GTP] <-> [Raf1-Ras-GTP]** | **K47=1.75** | **Kr47=0.05** | **(**[**Yamada et al, 2004**](#_ENREF_102)**)** |
| **r48** | **[Raf1-Ras-GTP] -> Raf1active + [Ras-GTP]** | **K48=0.7624** |  | **(**[**Yamada et al, 2004**](#_ENREF_102)**)** |
| **r49** | **Raf1active + MEK <-> [Raf1active-MEK]** | **K49=4** | **Kr49=0.01833** | **(**[**Huang & Ferrell, 1996**](#_ENREF_32)**;** [**Schoeberl et al, 2002**](#_ENREF_80)**;** [**Ung et al, 2008**](#_ENREF_92)**)** |
| **r50** | **[Raf1active-MEK] -> Raf1active + pMEK** | **K50=3.5** |  | **(**[**Huang & Ferrell, 1996**](#_ENREF_32)**;** [**Schoeberl et al, 2002**](#_ENREF_80)**;** [**Ung et al, 2008**](#_ENREF_92)**)** |
| **r51** | **Raf1active + pMEK <-> [Raf1active-pMEK]** | **K51=4** | **Kr51=0.01833** | **(**[**Huang & Ferrell, 1996**](#_ENREF_32)**;** [**Schoeberl et al, 2002**](#_ENREF_80)**;** [**Ung et al, 2008**](#_ENREF_92)**)** |
| **r52** | **[Raf1active-pMEK] -> Raf1active + ppMEK** | **K52=2.9** |  | **(**[**Huang & Ferrell, 1996**](#_ENREF_32)**;** [**Schoeberl et al, 2002**](#_ENREF_80)**;** [**Ung et al, 2008**](#_ENREF_92)**)** |
| **r53** | **ppMEK + ERK <-> [ppMEK-ERK]** | **K53=3** | **Kr53=0.033** | **(**[**Huang & Ferrell, 1996**](#_ENREF_32)**;** [**Schoeberl et al, 2002**](#_ENREF_80)**;** [**Ung et al, 2008**](#_ENREF_92)**)** |
| **r54** | **[ppMEK-ERK] -> ppMEK + pERK** | **K54=16** |  | **(**[**Huang & Ferrell, 1996**](#_ENREF_32)**;** [**Schoeberl et al, 2002**](#_ENREF_80)**;** [**Ung et al, 2008**](#_ENREF_92)**)** |
| **r55** | **ppMEK + pERK <-> [ppMEK-pERK]** | **K55=3** | **Kr55=0.033** | **(**[**Huang & Ferrell, 1996**](#_ENREF_32)**;** [**Schoeberl et al, 2002**](#_ENREF_80)**;** [**Ung et al, 2008**](#_ENREF_92)**)** |
| **r56** | **[ppMEK-pERK] -> ppMEK + ppERK** | **K56=5.7** |  | **(**[**Huang & Ferrell, 1996**](#_ENREF_32)**;** [**Schoeberl et al, 2002**](#_ENREF_80)**;** [**Ung et al, 2008**](#_ENREF_92)**)** |
| **r57** | **Raf1active + Pase <-> [Raf1active-Pase]** | **K57=71.7** | **Kr57=0.2** | **(**[**Huang & Ferrell, 1996**](#_ENREF_32)**;** [**Schoeberl et al, 2002**](#_ENREF_80)**;** [**Ung et al, 2008**](#_ENREF_92)**)** |
| **r58** | **[Raf1active-Pase] -> Raf1 + Pase** | **K58=1** |  | **(**[**Huang & Ferrell, 1996**](#_ENREF_32)**;** [**Schoeberl et al, 2002**](#_ENREF_80)**;** [**Ung et al, 2008**](#_ENREF_92)**)** |
| **r59** | **ppMEK + Pase2 <-> [ppMEK-Pase2]** | **K59=14.3** | **Kr59=0.8** | **(**[**Huang & Ferrell, 1996**](#_ENREF_32)**;** [**Schoeberl et al, 2002**](#_ENREF_80)**;** [**Ung et al, 2008**](#_ENREF_92)**)** |
| **r60** | **[ppMEK-Pase2] -> pMEK + Pase2** | **K60=0.058** |  | **(**[**Huang & Ferrell, 1996**](#_ENREF_32)**;** [**Schoeberl et al, 2002**](#_ENREF_80)**;** [**Ung et al, 2008**](#_ENREF_92)**)** |
| **r61** | **pMEK + Pase2 <-> [pMEK-Pase2]** | **K61=0.25** | **Kr61=0.5** | **(**[**Huang & Ferrell, 1996**](#_ENREF_32)**;** [**Schoeberl et al, 2002**](#_ENREF_80)**;** [**Ung et al, 2008**](#_ENREF_92)**)** |
| **r62** | **[pMEK-Pase2] -> MEK + Pase2** | **K62=0.58** |  | **(**[**Huang & Ferrell, 1996**](#_ENREF_32)**;** [**Schoeberl et al, 2002**](#_ENREF_80)**;** [**Ung et al, 2008**](#_ENREF_92)**)** |
| **r63** | **ppERK + Pase3 <-> [ppERK-Pase3]** | **K63=7** | **Kr63=0.6** | **(**[**Huang & Ferrell, 1996**](#_ENREF_32)**;** [**Schoeberl et al, 2002**](#_ENREF_80)**;** [**Ung et al, 2008**](#_ENREF_92)**)** |
| **r64** | **[ppERK-Pase3] -> pERK + Pase3** | **K64=0.27** |  | **(**[**Huang & Ferrell, 1996**](#_ENREF_32)**;** [**Schoeberl et al, 2002**](#_ENREF_80)**;** [**Ung et al, 2008**](#_ENREF_92)**)** |
| **r65** | **pERK + Pase3 <-> [pERK-Pase3]** | **K65=5** | **Kr65=0.5** | **(**[**Huang & Ferrell, 1996**](#_ENREF_32)**;** [**Schoeberl et al, 2002**](#_ENREF_80)**;** [**Ung et al, 2008**](#_ENREF_92)**)** |
| **r66** | **[pERK-Pase3] -> ERK + Pase3** | **K66=0.3** |  | **(**[**Huang & Ferrell, 1996**](#_ENREF_32)**;** [**Schoeberl et al, 2002**](#_ENREF_80)**;** [**Ung et al, 2008**](#_ENREF_92)**)** |
| **r67** | **ppERK + [pEGF-EGFR2-pShc-Grb2-SOS] <-> [ppERK-pEGF-EGFR2-pShc-Grb2-SOS]** | **K67=8.898** | **Kr67=1** | **(**[**Yamada et al, 2004**](#_ENREF_102)**)** |
| **r68** | **[ppERK-pEGF-EGFR2-pShc-Grb2-SOS] -> ppERK + [pEGF-EGFR2] + pShc + Grb2 + pSOS** | **K68=0.0426** |  | **(**[**Yamada et al, 2004**](#_ENREF_102)**)** |
| **r69** | **ppERK + [pEGF-EGFR2-Grb2-SOS] <-> [ppERK-pEGF-EGFR2-Grb2-SOS]** | **K69=8.898** | **Kr69=1** | **(**[**Yamada et al, 2004**](#_ENREF_102)**)** |
| **r70** | **[ppERK-pEGF-EGFR2-Grb2-SOS] -> ppERK + [pEGF-EGFR2] + Grb2 + pSOS** | **K70=0.0426** |  | **(**[**Yamada et al, 2004**](#_ENREF_102)**)** |
| **r71** | **pSOS -> SOS** | **K71=0.002** |  | **(**[**Sasagawa et al, 2005**](#_ENREF_79)**)** |
| **r72** | **ProEGFR -> EGFR** | **K72=0.005** |  | **(**[**Yamada et al, 2004**](#_ENREF_102)**)** |
| **r73** | **[pEGF-EGFR2-pShc-Grb2-SOS] + cbl <-> [pEGF-EGFR2-pShc-Grb2-SOS-cbl]** | **K73=0.5** | **Kr73=0.005** | **(**[**Yamada et al, 2004**](#_ENREF_102)**)** |
| **r74** | **[pEGF-EGFR2-pShc-Grb2-SOS-cbl] + EPn <-> [pEGF-EGFR2-pShc-Grb2-SOS-cbl-EPn]** | **K74=5** | **Kr74=0.1** | **(**[**Yamada et al, 2004**](#_ENREF_102)**)** |
| **r75** | **[pEGF-EGFR2-pShc-Grb2-SOS-cbl-EPn] -> cbl + [Grb2-SOS] + EPn + pShc** | **K75=0.001** |  | **(**[**Yamada et al, 2004**](#_ENREF_102)**)** |
| **r76** | **[pEGF-EGFR2-Grb2-SOS] + cbl <-> [pEGF-EGFR2-Grb2-SOS-cbl]** | **K76=0.5** | **Kr76=0.005** | **(**[**Yamada et al, 2004**](#_ENREF_102)**)** |
| **r77** | **[pEGF-EGFR2-Grb2-SOS-cbl] + EPn <-> [pEGF-EGFR2-Grb2-SOS-cbl-EPn]** | **K77=5** | **Kr77=0.1** | **(**[**Yamada et al, 2004**](#_ENREF_102)**)** |
| **r78** | **[pEGF-EGFR2-Grb2-SOS-cbl-EPn] -> cbl + [Grb2-SOS] + EPn** | **K78=0.001** |  | **(**[**Yamada et al, 2004**](#_ENREF_102)**)** |
| **r79** | **[pEGF-EGFR2] + cbl <-> [pEGF-EGFR2-cbl]** | **K79=0.5** | **Kr79=0.005** | **(**[**Sasagawa et al, 2005**](#_ENREF_79)**;** [**Yamada et al, 2004**](#_ENREF_102)**)** |
| **r80** | **[pEGF-EGFR2-cbl] + EPn <-> [pEGF-EGFR2-cbl-EPn]** | **K80=5** | **Kr80=0.1** | **(**[**Sasagawa et al, 2005**](#_ENREF_79)**;** [**Yamada et al, 2004**](#_ENREF_102)**)** |
| **r81** | **[pEGF-EGFR2-cbl-EPn] -> cbl + EPn** | **K81=0.001** |  | **(**[**Sasagawa et al, 2005**](#_ENREF_79)**;** [**Yamada et al, 2004**](#_ENREF_102)**)** |
| **r82** | **[pEGF-EGFR2] + PI3K <-> [pEGF-EGFR2-PI3K]** | **K82=14** | **Kr82=0.1743** | **(**[**Kiyatkin et al, 2006**](#_ENREF_41)**;** [**Ung et al, 2008**](#_ENREF_92)**)** |
| **r83** | **[pEGF-EGFR2-PI3K] <-> [pEGF-EGFR2-pPI3K]** | **K83=33.72** | **Kr83=0.000337** | **(**[**Kiyatkin et al, 2006**](#_ENREF_41)**;** [**Ung et al, 2008**](#_ENREF_92)**)** |
| **r84** | **[pEGF-EGFR2-PI3K] <-> [pEGF-EGFR2] + pPI3K** | **K84=0.09** | **Kr84=0.1764** | **(**[**Kiyatkin et al, 2006**](#_ENREF_41)**)** |
| **r85** | **TP4 + pPI3K <-> [TP4-pPI3K]** | **K85=1** | **Kr85=0.038** | **(**[**Kiyatkin et al, 2006**](#_ENREF_41)**)** |
| **r86** | **[TP4-pPI3K] -> [TP4-PI3K]** | **K86=0.595** |  | **(**[**Kiyatkin et al, 2006**](#_ENREF_41)**)** |
| **r87** | **[TP4-PI3K] <-> TP4 + PI3K** | **K87=4.7E-06** | **Kr87=2.3E-06** | **(**[**Kiyatkin et al, 2006**](#_ENREF_41)**)** |
| **r88** | **pPI3K + PIP2 <-> [pPI3K-PIP2]** | **K88=25** | **Kr88=3.5** | **(**[**Kiyatkin et al, 2006**](#_ENREF_41)**)** |
| **r89** | **[pPI3K-PIP2] -> pPI3K + PIP3** | **K89=25** |  | **(**[**Kiyatkin et al, 2006**](#_ENREF_41)**)** |
| **r90** | **Akt + PIP3 <-> Aktm** | **K90=10** | **Kr90=3** | **(**[**Kiyatkin et al, 2006**](#_ENREF_41)**)** |
| **r91** | **Aktm + PDK1 <-> [Aktm-PDK1]** | **K91=10** | **Kr91=1** | **(**[**Kiyatkin et al, 2006**](#_ENREF_41)**)** |
| **r92** | **[Aktm-PDK1] -> [pAktm-PDK1]** | **K92=10** |  | **(**[**Kiyatkin et al, 2006**](#_ENREF_41)**)** |
| **r93** | **[pAktm-PDK1] <-> pAktm + PDK1** | **K93=0.1** | **Kr93=0.005** | **(**[**Kiyatkin et al, 2006**](#_ENREF_41)**)** |
| **r94** | **pAktm <-> pAkt + PIP3** | **K94=1** | **Kr94=0.001** | **(**[**Kiyatkin et al, 2006**](#_ENREF_41)**)** |
| **r95** | **[pAkt-Takt] -> [Akt-Takt]** | **K95=0.05** |  | **(**[**Kiyatkin et al, 2006**](#_ENREF_41)**)** |
| **r96** | **[Akt-Takt] <-> Akt + Takt** | **K96=0.001** | **Kr96=0.001** | **(**[**Kiyatkin et al, 2006**](#_ENREF_41)**)** |
| **r97** | **pAkt + Takt <-> [pAkt-Takt]** | **K97=10** | **Kr97=1** | **(**[**Kiyatkin et al, 2006**](#_ENREF_41)**)** |
| **r98** | **pAktm + Takt <-> [pAktm-Takt]** | **K98=10** | **Kr98=1** | **(**[**Kiyatkin et al, 2006**](#_ENREF_41)**)** |
| **r99** | **[pAktm-Takt] -> [Aktm-Takt]** | **K99=0.05** |  | **(**[**Kiyatkin et al, 2006**](#_ENREF_41)**)** |
| **r100** | **[Aktm-Takt] <-> Aktm + Takt** | **K100=0.001** | **Kr100=0.001** | **(**[**Kiyatkin et al, 2006**](#_ENREF_41)**)** |
| **r101** | **[pAktm-PDK1] + Takt <-> [pAktm-PDK1-Takt]** | **K101=10** | **Kr101=1** | **(**[**Kiyatkin et al, 2006**](#_ENREF_41)**)** |
| **r102** | **[pAktm-PDK1-Takt] -> [Aktm-PDK1-Takt]** | **K102=0.05** |  | **(**[**Kiyatkin et al, 2006**](#_ENREF_41)**)** |
| **r103** | **[Aktm-PDK1-Takt] <-> [Aktm-PDK1] + Takt** | **K103=0.001** | **Kr103=0.001** | **(**[**Kiyatkin et al, 2006**](#_ENREF_41)**)** |
| **r104** | **Raf1active + pAkt_total -> pRaf1active + pAkt_total** | **Kon= 0.1** | **Km= 0.2** | **(**[**Kiyatkin et al, 2006**](#_ENREF_41)**)** |
| **r105** | **pRaf1active -> Raf1active** | **K105=1** |  | **(**[**Kiyatkin et al, 2006**](#_ENREF_41)**)** |
| **106** | **[pEGF-EGFR2] + STAT3c <-> [pEGF-EGFR2-STAT3c]** | **K106=5.5** | **Kr106=11.74** | **(**[**Hsieh et al, 2010**](#_ENREF_30)**),estimation** |
| **107** | **[pEGF-EGFR2-STAT3c] -> [pEGF-EGFR2] + pSTAT3c** | **K107=0.4** |  | **(**[**Yamada et al, 2003**](#_ENREF_101)**)** |
| **108** | **[pEGF-EGFR2] + pSTAT3c <-> [pEGF-EGFR2-pSTAT3c]** | **K108=5** | **Kr108=0.5** | **(**[**Yamada et al, 2003**](#_ENREF_101)**)** |
| **109** | **pSTAT3c + PP1 <-> [pSTAT3c-PP1]** | **K109=1** | **Kr109=0.2** | **(**[**Yamada et al, 2003**](#_ENREF_101)**)** |
| **110** | **[pSTAT3c-PP1] -> STAT3c + PP1** | **K110=0.003** |  | **(**[**Yamada et al, 2003**](#_ENREF_101)**)** |
| **111** | **pSTAT3c + pSTAT3c <-> [pSTAT3c-pSTAT3c]** | **K111=20** | **Kr111=0.1** | **(**[**Yamada et al, 2003**](#_ENREF_101)**)** |
| **112** | **[pSTAT3c-pSTAT3c] + PP1 <-> [pSTAT3c-pSTAT3c-PP1]** | **K112=1** | **Kr112=0.2** | **(**[**Yamada et al, 2003**](#_ENREF_101)**)** |
| **113** | **[pSTAT3c-pSTAT3c-PP1] -> [STAT3c-pSTAT3c] + PP1** | **K113=0.003** |  | **(**[**Yamada et al, 2003**](#_ENREF_101)**)** |
| **114** | **STAT3c + pSTAT3c <-> [STAT3c-pSTAT3c]** | **K114=0.0002** | **Kr114=0.2** | **(**[**Yamada et al, 2003**](#_ENREF_101)**)** |
| **115** | **[pSTAT3c-pSTAT3c] -> [pSTAT3n-pSTAT3n]** | **K115=0.005** |  | **(**[**Yamada et al, 2003**](#_ENREF_101)**)** |
| **116** | **pSTAT3n + pSTAT3n <-> [pSTAT3n-pSTAT3n]** | **K116=20** | **Kr116=0.1** | **(**[**Yamada et al, 2003**](#_ENREF_101)**)** |
| **117** | **[pSTAT3n-pSTAT3n] + PP2 <-> [pSTAT3n-pSTAT3n-PP2]** | **K117=1** | **Kr117=0.2** | **(**[**Yamada et al, 2003**](#_ENREF_101)**)** |
| **118** | **[pSTAT3n-pSTAT3n-PP2] -> [STAT3n-pSTAT3n] + PP2** | **K118=0.005** |  | **(**[**Yamada et al, 2003**](#_ENREF_101)**)** |
| **119** | **STAT3n + pSTAT3n <-> [STAT3n-pSTAT3n]** | **K119=0.0002** | **Kr119=0.2** | **(**[**Yamada et al, 2003**](#_ENREF_101)**)** |
| **120** | **pSTAT3n + PP2 <-> [pSTAT3n-PP2]** | **K120=1** | **Kr120=0.2** | **(**[**Yamada et al, 2003**](#_ENREF_101)**)** |
| **121** | **[pSTAT3n-PP2] -> STAT3n + PP2** | **K121=0.005** |  | **(**[**Yamada et al, 2003**](#_ENREF_101)**)** |
| **122** | **STAT3n -> STAT3c** | **K122=0.05** |  | **(**[**Yamada et al, 2003**](#_ENREF_101)**)** |
| **r123** | **PIP3 -> PIP2** | **K123=17** |  | **(**[**Yamada et al, 2003**](#_ENREF_101)**)** |
| **124** | **[pEGF-EGFR2-STAT3c] + cbl <-> [pEGF-EGFR2-STAT3c-cbl]** | **K124=0.5** | **Kr124=0.005** | **(**[**Yamada et al, 2004**](#_ENREF_102)**), estimation** |
| **125** | **[pEGF-EGFR2-STAT3c-cbl] + EPn <-> [pEGF-EGFR2-STAT3c-cbl-EPn]** | **K125=5** | **Kr125=0.1** | **(**[**Yamada et al, 2004**](#_ENREF_102)**) , estimation** |
| **126** | **[pEGF-EGFR2-STAT3c-cbl-EPn] -> STAT3c + cbl + EPn** | **K126=0.001** |  | **(**[**Yamada et al, 2004**](#_ENREF_102)**) , estimation** |
| **127** | **[pEGF-EGFR2-PI3K] + cbl <-> [pEGF-EGFR2-PI3K-cbl]** | **K127=0.5** | **Kr127=0.005** | **(**[**Yamada et al, 2004**](#_ENREF_102)**) , estimation** |
| **128** | **[pEGF-EGFR2-PI3K-cbl] + EPn <-> [pEGF-EGFR2-PI3K-cbl-EPn]** | **K128=5** | **Kr128=0.1** | **(**[**Yamada et al, 2004**](#_ENREF_102)**) , estimation** |
| **129** | **[pEGF-EGFR2-PI3K-cbl-EPn] -> PI3K + EPn** | **K129=0.001** |  | **(**[**Yamada et al, 2004**](#_ENREF_102)**) , estimation** |

**2- rules**

| **rule1** | **pAkt_total = pAkt + pAktm** | **Repeated Assignment** |
| --- | --- | --- |

**3- Nonzero species:**

| **Name** | **Concentration**  **(µM)** | **References** |
| --- | --- | --- |
| **EGF** | **0.008197** | **-** |
| **EGFR** | **0.3** | **(**[**Sasagawa et al, 2005**](#_ENREF_79)**)** |
| **cbl** | **0.8** | **(**[**Sasagawa et al, 2005**](#_ENREF_79)**)** |
| **Shc** | **1** | **(**[**Sasagawa et al, 2005**](#_ENREF_79)**)** |
| **SHP2** | **0.1** | **(**[**Sasagawa et al, 2005**](#_ENREF_79)**)** |
| **Grb2** | **1** | **(**[**Sasagawa et al, 2005**](#_ENREF_79)**)** |
| **SOS** | **0.3** | **(**[**Sasagawa et al, 2005**](#_ENREF_79)**)** |
| **Ras-GDP** | **0.15** | **(**[**Sasagawa et al, 2005**](#_ENREF_79)**)** |
| **Ras-GAP** | **0.1** | **(**[**Sasagawa et al, 2005**](#_ENREF_79)**)** |
| **Raf1** | **0.5** | **(**[**Sasagawa et al, 2005**](#_ENREF_79)**)** |
| **MEK** | **0.68** | **(**[**Sasagawa et al, 2005**](#_ENREF_79)**)** |
| **ERK** | **0.4** | **(**[**Sasagawa et al, 2005**](#_ENREF_79)**)** |
| **Pase** | **0.5** | **(**[**Sasagawa et al, 2005**](#_ENREF_79)**)** |
| **Pase2** | **0.02** | **(**[**Sasagawa et al, 2005**](#_ENREF_79)**)** |
| **Pase3** | **0.002** | **(**[**Sasagawa et al, 2005**](#_ENREF_79)**)** |
| **ProEGFR** | **1** | **(**[**Sasagawa et al, 2005**](#_ENREF_79)**)** |
| **EPn** | **0.5** | **(**[**Sasagawa et al, 2005**](#_ENREF_79)**)** |
| **PI3K** | **0.2** | **(**[**Kiyatkin et al, 2006**](#_ENREF_41)**)** |
| **TP4** | **0.2** | **(**[**Kiyatkin et al, 2006**](#_ENREF_41)**)** |
| **PIP2** | **0.5** | **(**[**Kiyatkin et al, 2006**](#_ENREF_41)**)** |
| **Akt** | **0.1** | **(**[**Kiyatkin et al, 2006**](#_ENREF_41)**)** |
| **PDK1** | **0.1** | **(**[**Kiyatkin et al, 2006**](#_ENREF_41)**)** |
| **Takt** | **0.1** | **(**[**Kiyatkin et al, 2006**](#_ENREF_41)**)** |
| **STAT3c** | **1** | **(**[**Yamada et al, 2003**](#_ENREF_101)**)** |
| **PP1** | **0.5** | **(**[**Yamada et al, 2003**](#_ENREF_101)**)** |
| **PP2** | **0.6** | **(**[**Yamada et al, 2003**](#_ENREF_101)**)** |
|  |  |  |

**1. Yamada, S., T. Taketomi, and A. Yoshimura, *Model analysis of difference between EGF pathway and FGF pathway.* Biochem Biophys Res Commun, 2004. 314(4): p. 1113-20.**

**2. Schoeberl, B., et al., *Computational modeling of the dynamics of the MAP kinase cascade activated by surface and internalized EGF receptors.* Nat Biotechnol, 2002. 20(4): p. 370-5.**

**3. Sasagawa, S., et al., *Prediction and validation of the distinct dynamics of transient and sustained ERK activation.* Nat Cell Biol, 2005. 7(4): p. 365-73.**

**4. Kholodenko, B.N., et al., *Quantification of short term signaling by the epidermal growth factor receptor.* J Biol Chem, 1999. 274(42): p. 30169-81.**

**5. Huang, C.Y. and J.E. Ferrell, Jr., *Ultrasensitivity in the mitogen-activated protein kinase cascade.* Proc Natl Acad Sci U S A, 1996. 93(19): p. 10078-83.**

**6. Ung, C.Y., et al., *Simulation of the regulation of EGFR endocytosis and EGFR-ERK signaling by endophilin-mediated RhoA-EGFR crosstalk.* FEBS Lett, 2008. 582(15): p. 2283-90.**

**7. Kiyatkin, A., et al., *Scaffolding protein Grb2-associated binder 1 sustains epidermal growth factor-induced mitogenic and survival signaling by multiple positive feedback loops.* J Biol Chem, 2006. 281(29): p. 19925-38.**

**8. Hsieh, M.Y., et al., *Spatio-temporal modeling of signaling protein recruitment to EGFR.* BMC Syst Biol, 2010. 4: p. 57.**

**9. Yamada, S., et al., *Control mechanism of JAK/STAT signal transduction pathway.* FEBS Lett, 2003. 534(1-3): p. 190-6.**

**Supplementary Table 2**

**NSCLC model:**

1. **Modified reactions**

Note: Michaelis Menten constants are given in µM, first order rate constants in s^-1^ and second order rate constants in µM^-1^ s^-1^

|  | **Reaction** | **Parameter** | | **reference** |
| --- | --- | --- | --- | --- |
| **r3** | **[EGF-EGFR2] -> [pEGF-EGFR2]** | **Vmax=0.24** | **Km= 13** | **[**[**1-3**](#_ENREF_1)**]** |
| **r73** | **[pEGF-EGFR2-pShc-Grb2-SOS] + cbl <-> [pEGF-EGFR2-pShc-Grb2-SOS-cbl]** | **K73=0.2** | **K73r=0.0025** | **Estimation** |
| **r74** | **[pEGF-EGFR2-pShc-Grb2-SOS-cbl] + EPn <-> [pEGF-EGFR2-pShc-Grb2-SOS-cbl-EPn]** | **K74=2** | **K74r=0.05** | **Estimation** |
| **r75** | **[pEGF-EGFR2-pShc-Grb2-SOS-cbl-EPn] -> cbl + [Grb2-SOS] + EPn + pShc** | **K75=0.0005** |  | **Estimation** |
| **r76** | **[pEGF-EGFR2-Grb2-SOS] + cbl <-> [pEGF-EGFR2-Grb2-SOS-cbl]** | **K76=0.2** | **K76r=0.0025** | **Estimation** |
| **r77** | **[pEGF-EGFR2-Grb2-SOS-cbl] + EPn <-> [pEGF-EGFR2-Grb2-SOS-cbl-EPn]** | **K77=2** | **K77r=0.05** | **Estimation** |
| **r78** | **[pEGF-EGFR2-Grb2-SOS-cbl-EPn] -> cbl + [Grb2-SOS] + EPn** | **K78=0.0005** |  | **Estimation** |
| **r79** | **[pEGF-EGFR2] + cbl <-> [pEGF-EGFR2-cbl]** | **K79=0.2** | **K79r=0.0005** | **Estimation** |
| **r80** | **[pEGF-EGFR2-cbl] + EPn <-> [pEGF-EGFR2-cbl-EPn]** | **K80=2** | **K80r=0.05** | **Estimation** |
| **r81** | **[pEGF-EGFR2-cbl-EPn] -> cbl + EPn** | **K81=0.0005** |  | **Estimation** |
| **r123** | **PIP3 -> PIP2 (deleted in loss of PTEN )** | **K123=17** |  | **Estimation** |
| **r124** | **[pEGF-EGFR2-STAT3c] + cbl <-> [pEGF-EGFR2-STAT3c-cbl]** | **K124=2** | **K124r=0.005** | **Estimation** |
| **r125** | **[pEGF-EGFR2-STAT3c-cbl] + EPn <-> [pEGF-EGFR2-STAT3c-cbl-EPn]** | **K125=2** | **K125r=0.05** | **Estimation** |
| **r126** | **[pEGF-EGFR2-STAT3c-cbl-EPn] -> STAT3c + cbl + EPn** | **K126=0.0005** |  | **Estimation** |
| **r127** | **[pEGF-EGFR2-PI3K] + cbl <-> [pEGF-EGFR2-PI3K-cbl]** | **K127=0.2** | **K127r=0.005** | **Estimation** |
| **r128** | **[pEGF-EGFR2-PI3K-cbl] + EPn <-> [pEGF-EGFR2-PI3K-cbl-EPn]** | **K128=2** | **K128r=0.05** | **Estimation** |
| **r129** | **[pEGF-EGFR2-PI3K-cbl-EPn] -> PI3K + EPn** | **K129=0.0005** |  | **Estimation** |

1. **Nonzero modified species**

| **EGFR** | **1** | **estimation** |
| --- | --- | --- |
| **Ras-GDP** | **0.3** | **Estimation** |
| **ERK** | **0.8** | **Estimation** |
| **PI3K** | **0.4** | **Estimation** |
| **Akt** | **0.2** | **Estimation** |
| **STAT3c** | **2** | **estimation** |

**1. Purvis, J., V. Ilango, and R. Radhakrishnan, *Role of network branching in eliciting differential short-term signaling responses in the hypersensitive epidermal growth factor receptor mutants implicated in lung cancer.* Biotechnol Prog, 2008. 24(3): p. 540-53.**

**2. Fan, Y.X., et al., *Ligand regulates epidermal growth factor receptor kinase specificity: activation increases preference for GAB1 and SHC versus autophosphorylation sites.* J Biol Chem, 2004. 279(37): p. 38143-50.**

**3. Brignola, P.S., et al., *Comparison of the biochemical and kinetic properties of the type 1 receptor tyrosine kinase intracellular domains. Demonstration of differential sensitivity to kinase inhibitors.* J Biol Chem, 2002. 277(2): p. 1576-85.**
