# Supplementary material for: Modeling of Tumor Progression in NSCLC and Intrinsic Resistance to TKI in Loss of PTEN Expression
Source: PLoS One. 2012 Oct 24;7(10):e48004. doi: 10.1371/journal.pone.0048004 (PMC3483873; doi:10.1371/journal.pone.0048004)
Supplement: Table S2 — NSCLC reactions and parameters. (DOCX) [file pone.0048004.s002.docx]

**Supplementary Table 2**

**NSCLC model:**

1. **Modified reactions**

Note: Michaelis Menten constants are given in µM, first order rate constants in s^-1^ and second order rate constants in µM^-1^ s^-1^

|  | **Reaction** | **Parameter** | | **reference** |
| --- | --- | --- | --- | --- |
| **r3** | **[EGF-EGFR2] -> [pEGF-EGFR2]** | **Vmax=0.24** | **Km= 13** | **[**[**1-3**](#_ENREF_1)**]** |
| **r73** | **[pEGF-EGFR2-pShc-Grb2-SOS] + cbl <-> [pEGF-EGFR2-pShc-Grb2-SOS-cbl]** | **K73=0.2** | **K73r=0.0025** | **Estimation** |
| **r74** | **[pEGF-EGFR2-pShc-Grb2-SOS-cbl] + EPn <-> [pEGF-EGFR2-pShc-Grb2-SOS-cbl-EPn]** | **K74=2** | **K74r=0.05** | **Estimation** |
| **r75** | **[pEGF-EGFR2-pShc-Grb2-SOS-cbl-EPn] -> cbl + [Grb2-SOS] + EPn + pShc** | **K75=0.0005** |  | **Estimation** |
| **r76** | **[pEGF-EGFR2-Grb2-SOS] + cbl <-> [pEGF-EGFR2-Grb2-SOS-cbl]** | **K76=0.2** | **K76r=0.0025** | **Estimation** |
| **r77** | **[pEGF-EGFR2-Grb2-SOS-cbl] + EPn <-> [pEGF-EGFR2-Grb2-SOS-cbl-EPn]** | **K77=2** | **K77r=0.05** | **Estimation** |
| **r78** | **[pEGF-EGFR2-Grb2-SOS-cbl-EPn] -> cbl + [Grb2-SOS] + EPn** | **K78=0.0005** |  | **Estimation** |
| **r79** | **[pEGF-EGFR2] + cbl <-> [pEGF-EGFR2-cbl]** | **K79=0.2** | **K79r=0.0005** | **Estimation** |
| **r80** | **[pEGF-EGFR2-cbl] + EPn <-> [pEGF-EGFR2-cbl-EPn]** | **K80=2** | **K80r=0.05** | **Estimation** |
| **r81** | **[pEGF-EGFR2-cbl-EPn] -> cbl + EPn** | **K81=0.0005** |  | **Estimation** |
| **r123** | **PIP3 -> PIP2 (deleted in loss of PTEN )** | **K123=17** |  | **Estimation** |
| **r124** | **[pEGF-EGFR2-STAT3c] + cbl <-> [pEGF-EGFR2-STAT3c-cbl]** | **K124=2** | **K124r=0.005** | **Estimation** |
| **r125** | **[pEGF-EGFR2-STAT3c-cbl] + EPn <-> [pEGF-EGFR2-STAT3c-cbl-EPn]** | **K125=2** | **K125r=0.05** | **Estimation** |
| **r126** | **[pEGF-EGFR2-STAT3c-cbl-EPn] -> STAT3c + cbl + EPn** | **K126=0.0005** |  | **Estimation** |
| **r127** | **[pEGF-EGFR2-PI3K] + cbl <-> [pEGF-EGFR2-PI3K-cbl]** | **K127=0.2** | **K127r=0.005** | **Estimation** |
| **r128** | **[pEGF-EGFR2-PI3K-cbl] + EPn <-> [pEGF-EGFR2-PI3K-cbl-EPn]** | **K128=2** | **K128r=0.05** | **Estimation** |
| **r129** | **[pEGF-EGFR2-PI3K-cbl-EPn] -> PI3K + EPn** | **K129=0.0005** |  | **Estimation** |

1. **Nonzero modified species**

| **EGFR** | **1** | **estimation** |
| --- | --- | --- |
| **Ras-GDP** | **0.3** | **Estimation** |
| **ERK** | **0.8** | **Estimation** |
| **PI3K** | **0.4** | **Estimation** |
| **Akt** | **0.2** | **Estimation** |
| **STAT3c** | **2** | **estimation** |

**1. Purvis, J., V. Ilango, and R. Radhakrishnan, *Role of network branching in eliciting differential short-term signaling responses in the hypersensitive epidermal growth factor receptor mutants implicated in lung cancer.* Biotechnol Prog, 2008. 24(3): p. 540-53.**

**2. Fan, Y.X., et al., *Ligand regulates epidermal growth factor receptor kinase specificity: activation increases preference for GAB1 and SHC versus autophosphorylation sites.* J Biol Chem, 2004. 279(37): p. 38143-50.**

**3. Brignola, P.S., et al., *Comparison of the biochemical and kinetic properties of the type 1 receptor tyrosine kinase intracellular domains. Demonstration of differential sensitivity to kinase inhibitors.* J Biol Chem, 2002. 277(2): p. 1576-85.**
